# A relative risk assessment of the open burning of WEEE

**DOI:** 10.1007/s11356-019-04282-3

**Published:** 2019-02-21

**Authors:** Alessandra Cesaro, Vincenzo Belgiorno, Giuliana Gorrasi, Gianluca Viscusi, Mentore Vaccari, Giovanni Vinti, Aleksander Jandric, Maria Isabel Dias, Andrew Hursthouse, Stefan Salhofer

**Affiliations:** 1 Sanitary Environmental Engineering Division (SEED), Department of Civil Engineering, University of Salerno, via Giovanni Paolo II, 84084 Fisciano, SA Italy; 2 Department of Industrial Engineering, University of Salerno, via Giovanni Paolo II, 84084 Fisciano, SA Italy; 3 Department of Civil, Environmental, Architectural Engineering and Mathematics, University of Brescia, via Branze 43, 25123 Brescia, Italy; 4 Waste Management Institute, BOKU University, Muthgasse 107, 1190 Vienna, Austria; 5 Centro de Ciências e Tecnologias Nucleares - C2TN, Campus Tecnológico e Nuclear, Polo de Loures, Instituto Superior Técnico, Estrada Nacional 10, km 139,7, Bobadela, 2696-066 Loures, Portugal; 6 University of the West of Scotland Paisley Campus, Paisley, PA1 2BE UK

**Keywords:** Air pollutants, Electronic waste, Environmental risk assessment, Informal recycling

## Abstract

Waste electric and electronic equipment (WEEE) represents a potential secondary source of valuable materials, whose recovery is a growing business activity worldwide. In low-income countries, recycling is carried out under poorly controlled conditions resulting in severe environmental pollution. High concentrations of both metallic and organic pollutants have been confirmed in air, soil, water, and sediments in countries with informal recycling areas. The release of these contaminants into the environment presents a risk to the health of the exposed population that has been widely acknowledged but still needs to be quantified. The aim of this work was to evaluate the relative risk from inhalation associated with the open burning of different kinds of WEEE. The shrinking core model was applied to estimate the concentration of the metals which would be released into the environment during the incineration of different types of WEEE. In addition, the potential generation of dioxins during the same informal practice was estimated, based on the plastic content of the WEEE. The results provided for the first time a comparative analysis of the risk posed from the open burning of WEEE components, proposing a methodology to address the absolute risk assessment to workers from the informal recycling of WEEE.

## Introduction

Waste electric and electronic equipment (WEEE) is regarded as a potential source of valuable elements. Depending on the specific devices, a wide range of concentrations of both precious metals and rare earth elements (REEs) can be present in addition to the prevailing base metals, such as copper and aluminum (Hagelüken 2006; Cui and Zhang 2008; Das et al. 2009; Binnemans et al. 2013; Ghosh et al. 2015; Cucchiella et al. 2015) along with varying quantities of plastics and inert fillers. However, many of the WEEE components are hazardous based on the concentrations of potentially harmful materials, both inorganic and organic. When the waste is improperly managed, these substances can be either directly released or act as precursor for the generation of further toxic by-products, which can pose a severe risk for both human health and the environment (Sepúlveda et al. 2010; Tsydenova and Bengtsson 2011; Chan and Wong 2013).

In the European Union, as well as in most high-income countries, WEEE is managed within strict legislative framework, implementing the extended producer responsibility (EPR) principle to promote separate collection, effective recycling as well as the development of eco-design of electric and electronic products. However, only one third of the WEEE produced is collected separately and destined for appropriate treatment. The remaining portion is likely to enter an informal management system (Tansel 2017). Some authors suggest that the difficult distinction between WEEE and UEEE (used electric and electronic equipment) accounts for the illegal transboundary movement of waste appliances (Zeng et al. 2013), mainly from high-income to developing countries. China has recently banned the import of 24 categories of waste, including post-consumer plastics and a range of hazardous residues. This decision has disrupted both the EU and US waste recycling industries which heavily rely on material export. It is widely anticipated that this will increase the environmental burdens associated with its management predominantly in the informal sector.

The informal recycling of WEEE refers to poorly regulated practices, which usually take place in either scattered workshops or domestic backyards in urban/suburban environments, with the main aim of recovering precious metals such as silver and gold (Tue et al. 2016; Ceballos and Dong 2016). To achieve this, devices are usually manually dismantled to separate the valuable components, reduced in size, and then subjected to basic treatment to liberate valuable materials. Different treatment methods can be applied, but the most frequently reported are acid leaching and open burning (Sepúlveda et al. 2010; Wang and Xu 2014). As all of these processes are performed under uncontrolled conditions, they may result in environmental emissions that, in turn, can pose severe risks to human health of both operators and wider public in the vicinity.

The contamination of air, soil, water, and sediments from informal WEEE recycling has been more recently documented (Tue et al. 2016; Alcántara-Concepción et al. 2016); high concentrations of both metallic and organic pollutants have been detected within the informal working sites (Grant et al. 2013) as well as spreading to the surrounding areas (Awasthi et al. 2016).

The release of contaminants in the environment can affect human health depending on both the specific composition of the WEEE material and the type of recycling practice. The former influences the mixture of contaminants that can enter the environment; the latter defines the physical state of the contaminant, determining its pathway. Some of the released contaminants are primary WEEE components (i.e., potentially toxic elements, flame retardants, ozone-depleting substances), whereas others are secondary products from combustion or released during chemical refining processes (Lundgren et al. 2012; Cayumil et al. 2016).

Both occupational and environmental exposure of humans to the pollutants during the informal treatment of WEEE has been described (Akormedi et al. 2013; Ohajinwa et al. 2017). According to these studies, the workers involved in the informal sector may be subjected to particularly dangerous exposure conditions. As they usually live either close to or within working sites, they may suffer additional exposure from the wider environmental contamination that pervades domestic environments (Bakhiyi et al. 2018).

Several field studies have been carried out to identify the extent of this type of contamination as well as to highlight the associated potential human burdens. The authors recently reported the evaluation of a strategy to identify the relative potential harm of different kinds of WEEE based on typical metal content and intrinsic hazard (Cesaro et al. 2018). It provided a semi-quantitative ranking of individual components, revealing significant differences in potential harm posed by different electronic appliances. While this is of value in designing management strategies, the health risk of an exposed target population has yet to be quantified.

The assessment of the risk as the probability that a specific contamination phenomenon can produce the loss of human life (Zhang et al. 2010) is difficult. The lack of comparable toxicity data for the contaminants potentially involved limits the absolute risk assessment. Moreover, the comprehensive characterization of e-waste contaminants as well as that of human exposure to alternative flame retardants still needs to be evaluated (Bakhiyi et al. 2018). These uncertainties directly affect the reliability of human health risk assessment for the informal treatment scenarios; however, they may be overcome when using a comparative risk characterization approach.

To investigate this potential, this study focuses on the assessment of the open burning of three WEEE components: (i) mobile printed circuit boards (PCBs), (ii) computer PCBs, and (iii) cables. The material composition of these components was identified from published data, and the operating conditions for open burning were reviewed to identify the most reliable exposure scenario to use in the relative risk assessment.

## The informal treatment of WEEE via open burning

Informal WEEE recycling is practiced in open air as well as in small workshops (Iqbal et al. 2015). The working environment is usually below an acceptable standard to provide basic occupational safety, and there are no proper sanitation conditions. Workers lack proper ventilation and lighting facilities and do not use adequate protective equipment, such as face and nose masks (Imran et al. 2017).

Among the techniques adopted, open burning is used for different purposes, including component separation, solder recovery from PCBs, and melting plastic components before open dumping as well as copper recovery from electric cables (Sepúlveda et al. 2010; Chan and Wong 2013). The latter, often performed in open pits and at relatively low temperatures, is one of the most commonly reported crude recycling practices (Perkins et al. 2014).

Open burning of WEEE has a direct environmental impact from the release of a number of harmful substances into the atmosphere: the deposition of the contaminants on soil, sediments, or water accounts for the indirect impact (Alcántara-Concepción et al. 2016). Residual ash washing into surface water results in additional water pollution. For example, Suzuki et al. (2016) evaluated the level of dioxin-like compounds in surface soils and river sediments collected in and around a WEEE processing village in northern Vietnam. Toxic equivalents in soils collected from the open-burning area for wires and cables had a median value of 13 pg/g for polychlorinated dibenzo-p-dioxins (PCDD) and 64 pg/g for polychlorinated dibenzofurans (PCDF), 4.8 pg/g for coplanar polychlorinated biphenyls (Co-PCBs), and 13 pg/g for polybrominated dibenzofurans (PCBDF). It is important to note that the median toxic equivalents in soils collected from open-burning sites tended to be 1 to 2 orders of magnitude higher than the median values for soils collected at least 100-m away, from footpaths in rice paddies in the same location (Suzuki et al. 2016).

The effects on human health arise either directly, during the recycling process, or indirectly, through the intake of contaminated water or via the contaminated food chain. However, air is the most important vector for the transport of hazardous contaminants during open burning of WEEE: Imran et al. (2017) observed that this practice produced fumes so dense that a wide area of the recycling location is affected, and both the informal workers and residents living close to the site have difficulty in breathing.

Such circumstances raise particular concern, especially when considering the intense working conditions experienced within the informal sector. In Pakistan, the informal working population, which is composed of both children and adults, normally works every day, for 10 to 12 h (Imran et al. 2017). In India, the burning of printed wiring boards (PWBs) usually operates 6 days a week, for 9 to 10 h a day (Steiner 2004).

In Agbogbloshie (Ghana), the situation is equally extreme: the informal workers, often children and adolescents, work for 10 to 12 h per day (Wittsiepe et al. 2015) and incessantly burn the wires and cables containing PVC (Fujimori et al. 2016). This results in the immediate environment being overwhelmed by thick black smoke, which takes a long time to clear (Asante et al. 2012; Itai et al. 2014; Wittsiepe et al. 2015).

In the informal WEEE recycling industry established in three Palestinian villages in south-west Hebron, open burning is most commonly used to extract valuable copper from plastic-insulated wires (Davis and Garb 2015). In this area, between 7 and 35 t of cables were reported to be burnt daily by “professional burners”, young men contracted by scrap yard owners to incinerate their cables. Professional burners often involve teenagers to assist them in burning the cables. Young people are not the sole vulnerable group involved: in China, children and pregnant women also take part in the removal of the plastic coating for wires (Song and Li 2014).

Being an informal activity, it is difficult to collect detailed information about the actual working conditions in the sector. Information is dispersed and definitions are not consistent, often referred to as “informal recycling of WEEE” or as “uncontrolled incineration practices.” However, some key characteristics can be defined: (i) the involvement of young people as informal workers and (ii) the long working period, ranging between 9 to 12 h/day, every day. In addition, the adverse health effects from the open burning of WEEE on people living in the surroundings of the informal workshops have been frequently reported in the literature (Tsydenova and Bengtsson 2011; Perkins et al. 2014).

## Materials and methods

The approach proposed was to identify the relative potential of different types of WEEE in producing adverse human health effects when undergoing open burning. To address this, data on the material composition of different end-of-life electronic items (mobile PCBs, computer PCBs, and wires) were obtained from scientific and technical literature studies.

Although limited in number, the information dealing with the concentration of both metals and plastic components for these items was subjected to an appropriate level of peer review/quality assurance (Cesaro et al. 2018), to provide the most reliable data set for the analysis.

### WEEE composition

#### Printed circuit boards from personal computers and mobile phones

The PCB is an ingenious design solution, which has enabled a very dense array of electronic components (e.g., switches, capacitors, diodes, etc.) to function in a highly limited space. PCBs provide mechanical support for the electronic components and secure their electrical connection using conductive etched tracks. Based on the number of layers of the conductive tracks, PCBs can be subdivided into three major groups: single-, double-, and multi-layered. With the addition of further conductive layers, it is possible to populate the PCBs more densely with electronic components (Yamane et al. 2011). However, for practical reasons in the recycling industry, the classification of PCBs is based on devices or group of devices from which the PCBs originate, e.g., from personal computers (PCs), from mobile phones, and from small household appliances.

Along with this universal application in technology, PCBs have also highly complex material composition. A single PCB can contain more than 40 different materials (Lu and Xu 2016). Materials contained in the PCBs can be generally divided into three groups: metals, plastics, and non-metallic inorganic substances. The plastics and inorganic plastic substances are generally identified in the scientific literature as a non-metal fraction (NMF) and make between 60–70 wt% of the PCBs. Metal contained in the PCBs ranges between 30–40 wt% of the PCBs (Zheng et al. 2009; Veit et al. 2014). However, unlike NMF fraction, which remains consistent across various types of PCBs, the metal content is highly dependent on the function of the device. For example, the mass share of the total metal fraction and the concentration of the most valuable metals, i.e., Cu and Au, is significantly higher in the PCBs originating from mobile phones than in those from PCs.

There are several types of PCB substrate currently in use, but approx. 70% of all types of PCBs have a FR-4 type of substrate. The FR-4 substrates, as classified by the National Electrical Manufacturers Association (NEMA), are made of multiple layers of laminate made of epoxy-reinforced resins. Furthermore, the FR-4 substrate is used where flame retardants are required. In general, the NMF of PCB consists of 65 wt% of glass fibers, 32 wt% epoxy resin, and < 3 wt% of impurities (Kumar et al. 2018).

Based on their economic value and their relative concentrations, the metals contained in PCB can be segregated into base metals, trace, and precious metals. The base metals mainly include Cu, Fe, Al, Pb, Sn, Zn, and Ni with concentration range between 25–30 wt% (Cu) down to 0.5–1 wt% (Ni or Zn). The trace and precious metals are present in concentrations between 1 and 20,000 ppm. The concentrations and the presence of trace and precious metals are significantly more volatile than that of base metals (Işıldar et al. 2016; Kaya 2016; Evangelopoulos et al. 2017).

Data on the material composition of PCBs from PCs and mobile phones are summarized in Tables 1 and 2, respectively.

**Table 1:** Selected elemental composition of PCBs from PCs

Base metals (%)							Trace and precious metals (ppm)										Reference
Cu	Fe	Al	Pb	Sn	Zn	Ni	Ba	Bi	Cr	Co	Ba	Sr	Ta	Pd	Au	Ag	
10.0	N/A	7.0	1.2	N/A	1.6	0.9	N/A	N/A	N/A	N/A	N/A	N/A	N/A	N/A	300	100	Zhang and Forssberg 1997
26.0	16.0	10.5	7.7	N/A	1.5	2.4	N/A	N/A	N/A	N/A	N/A	N/A	N/A	N/A	N/A	15,020	Williams 2010
20.2	7.3	5.7	5.5	8.8	4.5	0.4	N/A	N/A	N/A	N/A	N/A	N/A	N/A	N/A	1300	1600	Yamane et al. 2011
20.0	1.3	1.8	2.3	1.8	0.3	N/A	1900	50	N/A	48	11	380	7	150	240	570	Oguchi et al. 2011
33.0	1.8	1.5	1.3	3.5	0.5	N/A	19,000	440	N/A	280	140	430	2600	300	1500	3800	Oguchi et al. 2011
19.2	1.1	4.0	0.4	0.7	0.8	0.2	0.36	N/A	0.12	N/A	N/A	N/A	N/A	27	130	704	Behnamfard et al. 2013
18.5	2.1	1.3	2.7	4.9	N/A	0.4	N/A	N/A	N/A	N/A	N/A	N/A	N/A	97	86	694	Yazici and Deveci 2013
20.0	1.9	4.0	2.3	3.5	1.2	0.4	1900	240	0.12	164	75.5	405	1303.5	123.5	270	704	Median value

**Table 2:** Elemental composition of PCBs from mobile phones

Base metals (%)							Precious metals (ppm)		Reference
Cu	Fe	Al	Pb	Sn	Zn	Ni	Au	Ag	
32.3	0.5	1.8	0.3	–	0.1	0.7	30	4120	Williams 2010
35.1	–	–	2.7	4.0	–	–	1200	–	Kim et al. 2011
39.6	1.4	0.3	1.2	2.1	3.4	3.4	600	600	Kasper et al. 2011
38.3	6.5	1.0	1.3	3.1	1.0	1.7	1000	600	Kasper et al. 2011
37.8	4.9	0.6	1.2	2.6	1.8	2.5	900	500	Kasper et al. 2011
39.9	–	–	–	–	0.5	0.4	1	1	Jing-ying et al. 2012
24.2	0.2	3.3	0.9	1.4	0.1	0.3	600	1000	Ortuño et al. 2013
24.2	0.2	3.3	0.9	1.4	0.1	0.3	600	600	Median value

#### Cables and wiring

Rapid development and accessibility of the electrical and electronic equipment (EEE) are associated with the increased production of cables and wires. However, the recycling of cables and wires, due to their varying size and diverse applications, is particularly challenging. The structure of cables and wires is independent of their function: a conductive metal core for transmission of electricity and data usually made of high purity copper, an insulating layer, and a flame-retardant containing protection layer (Suresh et al. 2017). An overview of material composition of several types of cables is provided in Table 3.

**Table 3:** An overview of material composition of several types of Cu-core cables (Hischier et al. 2007)

Cable type	Component	Material	(g)	wt%
Computer power cable—1 conducting wire (without plugs, 10-cm length of cable)	Conductor material	Cu	1.29	19.88%
	Insulation	TPE elastomer	1.95	30.05%
	Black jacket	PVC	3.25	50.08%
Network cable—8 conducting wires (without plugs, 10-cm length of cable)	Conductor material	Cu	5	43.84%
	Insulation	PVC	5	43.84%
	Foil around insulation layer	PE	0.1	0.88%
	Shielding braid	Cu	1.2	10.52%
	Soft jacket	PVC	0.105	0.92%
Printer cable—25 conducting wires (without plugs, 10-cm length of cable)	Conductor material	Cu	4.6	47.17%
	Insulation	PVC	4,6	47.17%
	Fine copper wire to absorb noise	Cu	0.12	1.23%
	Fine aluminum layer	Al	0.09	0.92%
	Soft jacket	PVC	0.341	3.50%
Ribbon cable—20 conducting wires (with plugs, 1 kg of cable)	Conductor material	Cu	155	15.50%
	Cable jacket	PVC	155	15.50%
	Plugs (both ends)	HDPE	687	68.70%
	Contacts	brass	3	0.30%

### The shrinking Core model (SCM)

For the purposes of the relative risk assessment, the concentration of metals emitted from the open burning of WEEE was estimated by applying the shrinking core model (SCM). The data dealing with the concentration of metals in air, as reported in scientific literature, are not specifically associated with the open burning practices but a general reference to informal recycling of WEEE. The proportion of different categories of WEEE destined to this practice is not provided, so that linking the metal concentration in air to a fully characterized WEEE category is not possible. Thermodynamic simulations have also been performed (Dong et al. 2015; Yu et al. 2016), but the experimental conditions adopted do not reflect the uncontrolled situation of open burning.

The SCM is widely used to describe fluid–solid reactions that result in the shrinkage of the solid particles. It can apply to different areas, including pharmacokinetics, extractive metallurgy, control of gaseous pollutants, and catalyst regeneration (Gbor and Jia 2004; Fogler 2016). Further applications dealt with adsorption reactions: Fan et al. (2001) used the SCM to describe the behavior of a fixed-bed reactor during the reaction between gas phase H_2_S and perovskite-type sorbents: Jena et al. (2003) developed a SCM-based mass transfer formulation for batch adsorption processes. In the field of combustion reactions, the SCM is the standard theoretical framework (Sadhukhan et al. 2010; Buckmaster and Jackson 2013; Zhao et al. 2015, 2018; Wang et al. 2016) and it was used, in this work, to model the chemical reaction occurring during the open burning of WEEE.

This can be regarded as a heterogeneous reaction in which a gas, namely the ambient air, surrounds a solid particle and reacts with it. Such reactions are generally represented as follows:

1 $$ a{A}_{(s)}+b{B}_{(g)}\to c{C}_{(s)}+d{D}_{(g)} $$

The heterogeneous reactions of solid particles surrounded by a gaseous film can be described by the SCM, assuming that the reaction occurs first at the outer skin of the particle. The reaction zone then moves into the solid, leaving behind completely converted material and inert solid, referred to as ash, so that at any time, there exists an unreacted core of material which shrinks in size during the reaction (Levenspiel 1999). In accordance with the SCM, the reaction can be regarded as the succession of five steps (Levenspiel 1999):

1. Diffusion of the gaseous reactant through the film surrounding the particle to its solid surface;
2. Penetration and diffusion of the gas through the blanket of ash to the surface of the unreacted core;
3. Reaction of the gas with the solid;
4. Diffusion of the gaseous products through the ash back to the exterior surface of the solid;
5. Diffusion of the gaseous products through the gas film back into the main body of the fluid.

For the purposes of this work, the second step can be considered the rate-controlling one. In a gas/solid system such as for combustion, the shrinkage of the unreacted core is indeed much slower than the flow rate of the gas diffusing towards the unreacted core, so that it is possible to consider the shrinking process as being stationary. In this hypothesis, the gas flow within the ash layer can be expressed by the Fick’s law, according to the following expression:

2 $$ -\frac{1}{S_{\mathrm{ex}}}\frac{\mathrm{dN}}{\mathrm{dt}}=\mathcal{D}\frac{\mathrm{dC}}{\mathrm{dr}}=\mathrm{costant} $$

where

*S*_ex_: is the unchanging exterior surface of the solid particle;
*N*: is the number of moles of the gaseous reactant;
*D*_*e*_: is the effective diffusion coefficient of the gaseous reactant in the ash layer, evaluated considering the porosity and the tortuosity of the solid material;
*C*: is the concentration of gaseous reactant computed at standard conditions (298.15 K and 101 kPa).

Therefore, assuming that the solid particles involved in the reaction have a spherical shape, the conversion process develops as described by Eq. (2), meaning that the rate of reaction at any instant is given by its rate of diffusion to the reaction surface.

Considering that the mass (m) of a spherical particle is related to the density of its composing material (*ρ*) by the following expression:

3 $$ m=\frac{4}{3}\pi {r}^3\rho $$

Equation (1) can be also written as follows:

4 $$ \left(-\frac{dN}{dt}\right)\ast \frac{dm}{m^{\frac{4}{3}}}={\mathcal{D}}_{\mathrm{e}}\ast 16{\pi}^2\rho {\left(\frac{3}{4\pi \rho}\right)}^{\frac{4}{3}}\ast \mathrm{dc} $$

The solution to this equation is given by the following expression:

5 $$ \left(-\frac{dN}{dt}\right)\ast \left(\frac{1}{m^{\frac{1}{3}}}-\frac{1}{M^{\frac{1}{3}}}\right)={\mathcal{D}}_{\mathrm{e}}\ast \frac{16}{3}{\pi}^2\rho {\left(\frac{3}{4\pi \rho}\right)}^{\frac{4}{3}}\ast {c}_{ag} $$

where

*m*: is the mass of the solid particle;
*M*: is the initial mass of the solid particle;
*c*_ag_: is the bulk concentration of gaseous reactant evaluated.

In order to describe the heterogeneous reaction more realistically, it should be considered that as the solid particle core shrinks, the ash layer becomes thicker, slowing the rate of diffusion of the gas. According to the stoichiometry of a generic chemical reaction:

6 $$ \left(-\mathrm{d}{\mathrm{N}}_a\right)=\frac{a}{b}\left(-\mathrm{d}{\mathrm{N}}_b\right)=-\frac{a}{b}\frac{\mathrm{dm}}{\mathrm{MW}} $$

where

*a* and *b*: are the stoichiometric coefficients of the reactants;
*M*_W_: is the molecular weight of the solid reactant.

Considering this equivalence, Eq. (5) is solved using the following expression:

7 $$ {M}^{\frac{2}{3}}\left\{\left[\left(1-\left(\frac{m}{M}\right)\right)\right]-1,5\bullet \left[1-{\left(\frac{m}{M}\right)}^{\frac{2}{3}}\right]\right\}=-7.795\ast \frac{\mathrm{Mw}\ast b\ast {\mathcal{D}}_{\mathrm{e}}\ast {c}_{\mathrm{ag}}}{a\ast {\rho}^{\frac{1}{3}}}\ast t $$

In order to reduce the complexity of the mathematical model, an operating temperature of 550°C (823.15 K) was chosen based on previous studies (Gullett et al. 2007; Zhang et al. 2015), and the mass (mg) of ash produced after 1 h combustion of 1 t of WEEE components was obtained for the selected metals, to allow an estimate of the corresponding concentration in air (shown in Table 4).

**Table 4:** Mass of metallic ash and corresponding air concentrations from 1-h open burning of WEEE

Metal	Concentration (μg/m3)		
	Computer PCB	Mobile phone PCB	Wires and cables
Copper (Cu)	540.26	1021.65	1194.22
Lead (Pb)	82.76	43.18	–
Chromium (Cr)	0.15	–	–
Zinc (Zn)	22.05	13.46	–
Nickel (Ni)	5.43	14.91	–
Aluminum (Al)	29.71	18.98	–
Cobalt (Co)	0.24	–	–
Barium (Ba)	6.16	–	–
Strontium (Sr)	0.57	–	–

### The relative risk assessment

It was possible to estimate the concentration of metals released from the open burning of different types of WEEE, assuming a working time of 10 h.

For organic pollutants, the emitted concentrations in air were estimated on the basis of estimates previously reported in scientific literature (Gullett et al. 2007; Moltó et al. 2011; Zhang et al. 2015).

The emitted concentration of the i-th contaminant was used to estimate the exposure concentration (EC_I_), as described in the following equation:

8 $$ {\mathrm{EC}}_{\mathrm{I}}=\frac{C_{\mathrm{i}}\bullet \mathrm{ET}\bullet \mathrm{EF}\bullet \mathrm{ED}}{\mathrm{AT}} $$

where

*C*_I_: is the emitted concentration of the i-th contaminant (mg/m^3^);
ET: is the exposure time (hour/day);
EF: is the exposure frequency (day/year);
ED: is the exposure duration (years);
heterogeneous reactions AT: is the average time of exposure in a lifetime.

The non-cancer risk from the inhalation of the i-th contaminant, namely the hazard index (HI_I_), was calculated as follows:

9 $$ {\mathrm{HI}}_{\mathrm{I}}=\frac{{\mathrm{EC}}_{\mathrm{I}}}{\mathrm{RfC}} $$

where RfC is the inhalation reference concentration of the i-th contaminant (mg/m^3^).

The RfC values, defined as an estimate of a concentration under continuous exposure for individuals that does not present any risk of deleterious effects during a lifetime, were selected from international databases. For inorganic compounds, these values refer to the elemental metal or, if not available, to a metal compound that is likely to be produced during open burning, as highlighted in Table 5.

**Table 5:** Reference concentrations for inhalation of the contaminants of interest

Contaminant	RfC (μg/m3)	Reference
Copper (Cu)	140	ISS 2015*
Lead (Pb)	0.2	US-EPA 2017
Chromium (Cr)	0.1	US-EPA 2017
Zinc (Zn)	1050	ISS 2015*
Nickel (Ni)	0.014	US-EPA 2017
Aluminum (Al)	5	US-EPA 2017
Cobalt (Co)	0.006	ISS 2015*
Barium (Ba)	0.5	US-EPA 2017
Strontium (Sr)	0.2	US-EPA 2017
2,3,7,8-TCDD	0.00004	US-EPA 2017

*Obtained from the data provided for oral exposure by the US-EPA—Region 9 (2015). Environmental protection agency, toxicity, and chemical/physical properties for regional screening level (RSL) of chemical contaminants at superfund sites (http://www.epa.gov/region9/superfund/prg/)

For each WEEE component, the total hazard index (HI) was obtained as the sum of the inhalation hazard index estimated for the single contaminants.

The comparative analysis of the HI of the selected WEEE components was referred to a normalized HI (DpHI), which was calculated as the ratio between the HI of the single component and the minor HI.

## Results and discussion

The relative risk assessment for the open burning of computer PCBs, mobile phone PCBs, and cables is based on the comparison of the potential HI (Table 6), calculated for an exposure scenario defined by the literature review.

**Table 6:** Relative potential Hazard Index (pHI) for the combustion of selected WEEE

Contaminant	pHI		
	PC PCB	Mobile PCB	Wires
Copper (Cu)	1.6	3.0	3.5
Lead (Pb)	170.0	88.7	–
Chromium (Cr)	0.6	–	–
Zinc (Zn)	0.0	0.0	–
Nickel (Ni)	159.5	437.7	–
Aluminum (Al)	2.4	1.6	–
Cobalt (Co)	16.4	–	–
Barium (Ba)	5.1	–	–
Strontium (Sr)	1.2	–	–
2,3,7,8-TCDD	616.4	308.2	3082.2
*pHITOT*	973.3	839.2	3085.7
*∆* *pHITOT*	1.2	1.0	3.7

The potential hazard index (pHI) indicates the potential hazard posed by the uncontrolled incineration of a selected WEEE component. For the hazard to be acceptable, the pHI should be lower than 1: this would indicate that each contaminant is emitted in air at a concentration that is below the threshold limit represented by the corresponding reference concentration for inhalation.

As anticipated, this index is significantly higher than 1 for the WEEE components considered and is predominantly driven by the presence of the chlorine-containing plastics, which is expected to generate concentrations of 2,3,7,8-tetrachlorodibenzo-p-dioxin (2,3,7,8-TCDD) in air ranging between 0.03 and 0.3 μg/m^3^. These values, which are consistent with field studies at informal WEEE processing sites (Li et al. 2007; Wong et al. 2007; Tsydenova and Bengtsson 2011) are much lower than those estimated for inorganic pollutants. However, as 2,3,7,8-TCDD is a highly toxic compound, its reference concentration for inhalation can be up to approximately 10 times lower than those for the inorganic compounds.

It is worth pointing out that the pHI_2,3,7,8-TCDD_ for cables is one order of magnitude higher than that of the same organic compound from the open burning of both mobile and computer PCBs. This outcome depends, in turn, on the expected concentration in air of this pollutant. Gullett et al. (2007) characterized both air emission and residual ash produced during the simulated open burning of both circuit boards and insulated wires. The emissions from the latter were exceptionally high, even higher than the ones reported in previous studies. The authors attributed this result to the high concentration of chlorine-containing PVC insulation on the wires as well as by other factors related to the incomplete combustion. In contrast to the thermal treatment performed in industrialized facilities, open burning develops under uncontrolled conditions of temperature and oxygen supply, which do not promote complete combustion reactions and, in turn, results in the production of undesired pollutants in the exhaust gases. Combustion temperature, in particular, plays a key role in the formation of dioxins from the incineration of waste materials. Shibamoto et al. (2007) pointed out that dioxin formation occurs at temperatures above 450 °C and decreases significantly at temperatures above 850 °C, which are not likely to be reached in the proposed open burning scenario.

In the case of inorganic components, the results of the SCM highlight copper to have the highest concentration in air (540.26 and 1021.65 μg/m^3^ for computer and mobile PCBs, respectively), followed by lead (82.76 and 43.18 μg/m^3^ for computer and mobile PCBs, respectively) and aluminum (29.78 and 18.98 μg/m^3^ for computer and mobile PCBs, respectively). The differences are related to the initial mass of metals in each WEEE component, but the estimated values are consistent with those reported from field studies (Deng et al. 2006; Wong et al. 2007; Tsydenova and Bengtsson 2011), providing independent verification of the model predictions.

In the previously reported case studies, the values measured were lower, probably due to the location where the sampling operations were performed. Some data relate to air samples collected on the roof of a three-story building located on a street where open burning was performed together with other kinds of informal recycling practices (Deng et al. 2006; Wong et al. 2007): another study considers a sampling station situated on a building (Li et al. 2007). In these cases, the air samples were not taken in direct contact with the source of gaseous emission or even close to occupational exposure conditions.

However, the potential hazard associated to the single inorganic contaminant depends on its specific chemical species, its concentration in air, and its toxicity. In this view, it is possible to observe that the pHI for copper, although exceeding the threshold limit value for the hazard to be acceptable, is in the same order of magnitude, ranging between 1.6 and 3.5. Similar comparisons can be made for aluminum, whereas nickel greatly contributes to the overall potential hazard of the considered WEEE component, due to its higher toxicity.

The pHI calculation does not provide an absolute assessment of the risks for human health. A number of uncertainties affect the characterization of the contamination source, namely the air mass intercepted during the pollutant release from the WEEE incineration process. One of the main issues is related to the composition of the WEEE mass destined for open burning. The WEEE flow ending in the informal sector may contain a wide range of discharged appliances, and it is not possible to know the share of each WEEE component from the total amount of electronic waste sent for uncontrolled management. This circumstance limits the definition of WEEE samples representative of actual conditions in simulated open burning, which, in turn, provides unreliable assessment if it is intended to quantify the risks to human health. For this purpose, field measurement could be more effective but, in this case, the detected concentration of target pollutants in air cannot be directly associated with the presence of a specific WEEE component in the waste mass being incinerated, limiting the possible identification of prioritization strategies in the informal management of WEEE.

However, the comparative analysis can provide a relative risk factor for the incineration process by reference to the pHI. The DpHI represents the risk to human health associated with the open burning of selected WEEE components. It builds on previous work evaluating material balance (Cesaro et al. 2018) and refining it to accommodate management practices towards a viable model for risk assessment of open burning. This would provide direct support for decision-making when handling different WEEE streams.

The main findings of this study are that the potential risk for human health during the open burning of cables is much higher than that of computer PCBs, which is in turn higher than that for mobile phone PCBs.

The informal recycling of cables via uncontrolled incineration should therefore be prioritized when setting up strategies to improve the sustainability of WEEE processing. From a health risk analysis, it is not the presence of metals but the plastic components, especially those containing chlorine, which can act as precursor of organic compounds much more toxic than the inorganic ones. This confirms the urgent need for further studies to characterize, by both their chemical and toxicological properties, new persistent compounds that are being used as alternatives to conventional flame retardants, and/or plasticizers as well as emerging dioxin-related compounds which have recently been detected in the soil from around incineration sites in Ghana (Tue et al. 2016). Similarly, novel-brominated flame retardants have been identified in food samples grown near informal waste processing sites at higher levels than those obtained for samples in control sites in China (Labunska et al. 2015). These data, in turn, point to the need for better characterization of WEEE material composition.

The availability of data for the comprehensive characterization of the gaseous emission from the open burning of WEEE would greatly improve the effectiveness of using a risk-based procedure to prioritize improvements in waste management activity. Widening this approach to other types of WEEE as well as to other uncontrolled practices would support the integration of the informal recycling sector within the formal waste management sector and improve the sustainability of WEEE management in low-income regions. It is therefore important to recognize the need to frame the outcomes of a risk-based procedure within the socio-economic conditions of different areas as well as to integrate them with the interests of all relevant parties highlighted by Stewart and Hursthouse (2018). This would indeed provide a path to consensus and help to ensure the sustainability of any adopted WEEE management strategy to reduce the burdens on both human and environmental health.

## Conclusions

The open burning of WEEE is an informal recycling practice, widely applied in numerous low-income regions. Although the generation of metallic dusts and dioxins as well as their release in open air has been discussed in the literature, an approach to quantify the risks from the inhalation of these pollutants, especially by the informal workers, has yet to be proposed. This study identified and evaluated a comparative assessment of the potential risk associated to the open burning of different types of WEEE components. The relative risk assessment results show that there is considerable variation in risk from different components which should drive strategies to improve waste management and public health in affected regions.

A number of uncertainties have been identified, so that further research is needed to improve the potential of this method in driving field studies to develop absolute risk assessment as well as in raising awareness of the actual burdens on human health from the open burning of WEEE.

The modeling of this type of risk-based approach accommodating country-specific conditions as well as the integration of its outcomes with the needs of the different stakeholders holds the key to developing appropriate technical solutions.

## References

[CR1] Akormedi M, Asampong E, Fobil JN (2013). Working conditions and environmental exposures among electronic waste workers in Ghana. Int J Occup Environ Health.

[CR2] Alcántara-Concepción V, Gavilán-García A, Gavilán-García IC (2016). Environmental impacts at the end of life of computers and their management alternatives in México. J Clean Prod.

[CR3] Asante KA, Agusa T, Biney CA, Agyekum WA, Bello M, Otsuka M, Itai T, Takahashi S, Tanabe S (2012). Multi-trace element levels and arsenic speciation in urine of e-waste recycling workers from Agbogbloshie, Accra in Ghana. Sci Total Environ.

[CR4] Awasthi AK, Zeng X, Li J (2016). Environmental pollution of electronic waste recycling in India: a critical review. Environ Pollut.

[CR5] Bakhiyi B, Gravel S, Ceballos D, Flynn MA, Zayed J (2018). Has the question of e-waste opened a Pandora’s box? An overview of unpredictable issues and challenges. Environ Int.

[CR6] Behnamfard A, Salarirad MM, Vegliò F (2013). Process development for recovery of copper and precious metals from waste printed circuit boards with emphasize on palladium and gold leaching and precipitation. Waste Manag.

[CR7] Binnemans K, Jones PT, Blanpain B, van Gerven T, Yang Y, Walton A, Buchert M (2013). Recycling of rare earths: a critical review. J Clean Prod.

[CR8] Buckmaster J, Jackson TL (2013). An examination of the shrinking-core model of sub-micron aluminum combustion. Combust Theory Model.

[CR9] Cayumil R, Khanna R, Rajarao R, Mukherjee PS, Sahajwalla V (2016). Concentration of precious metals during their recovery from electronic waste. Waste Manag.

[CR10] Ceballos DM, Dong Z (2016). The formal electronic recycling industry: challenges and opportunities in occupational and environmental health research. Environ Int.

[CR11] Cesaro A, Belgiorno V, Vaccari M, Jandric A, Chung TD, Dias MI, Hursthouse A, Salhofer S (2018). A device-specific prioritization strategy based on the potential for harm to human health in informal WEEE recycling. Environ Sci Pollut Res.

[CR12] Chan JKY, Wong MH (2013). A review of environmental fate, body burdens, and human health risk assessment of PCDD/Fs at two typical electronic waste recycling sites in China. Sci Total Environ.

[CR13] Cucchiella F, D’Adamo I, Lenny Koh SC, Rosa P (2015). Recycling of WEEEs: an economic assessment of present and future e-waste streams. Renew Sust Energ Rev.

[CR14] Cui J, Zhang L (2008). Metallurgical recovery of metals from electronic waste: a review. J Hazard Mater.

[CR15] Das A, Vidyadhar A, Mehrotra SP (2009). A novel flowsheet for the recovery of metal values from waste printed circuit boards. Resour Conserv Recycl.

[CR16] Davis J-M, Garb Y (2015). A model for partnering with the informal e-waste industry: rationale, principles and a case study. Resour Conserv Recycl.

[CR17] Deng WJ, Louie PKK, Liu WK, Bi XH, Fu JM, Wong MH (2006). Atmospheric levels and cytotoxicity of PAHs and heavy metals in TSP and PM2.5 at an electronic waste recycling site in southeast China. Atmos Environ.

[CR18] Dong J, Chi Y, Tang Y, Ni M, Nzihou A, Weiss-Hortala E, Huang Q (2015). Partitioning of heavy metals in municipal solid waste pyrolysis, gasification, and incineration. Energy Fuel.

[CR19] Evangelopoulos P, Kantarelis E, Yang W (2017). Experimental investigation of pyrolysis of printed circuit boards for energy and materials recovery under nitrogen and steam atmosphere. Energy Procedia.

[CR20] Fan Y, Rajagopalan V, Suares GE, Amiridis MD (2001). Use of an “unreacted shrinking core” model in the reaction of H_2_S with perovskite-type sorbents. Ind Eng Chem Res.

[CR21] Fogler HS (2016) Elements of Chemical Reaction Engineering (5th Edition) chapter 14

[CR22] Fujimori T, Itai T, Goto A, Asante KA, Otsuka M, Takahashi S, Tanabe S (2016). Interplay of metals and bromine with dioxin-related compounds concentrated in e-waste open burning soil from Agbogbloshie in Accra, Ghana. Environ Pollut.

[CR23] Gbor PK, Jia CQ (2004). Critical evaluation of coupling particle size distribution with the shrinking core model. Chem Eng Sci.

[CR24] Ghosh B, Ghosh MK, Parhi P, Mukherjee PS, Mishra BK (2015). Waste printed circuit boards recycling: an extensive assessment of current status. J Clean Prod.

[CR25] Grant K, Goldizen FC, Sly PD, Brune MN, Neira M, van den Berg M, Norman RE (2013). Health consequences of exposure to e-waste: a systematic review. Lancet Glob Health.

[CR26] Gullett BK, Linak WP, Touati A, Wasson SJ, Gatica S, King CJ (2007). Characterization of air emissions and residual ash from open burning of electronic wastes during simulated rudimentary recycling operations. J Mater Cycles Waste Manag.

[CR27] Hagelüken C (2006). Recycling of electronic scrap at Umicore precious metals refining. Acta Metall Slovaca.

[CR28] Hischier R, Classen M, Lehmann M, Scharnhorst W (2007). Life Cycle Inventories of Electric and Electronic Equipment: Production, Use and Disposal, Ecoinvent.

[CR29] Imran M, Haydar S, Kim J, Awan MR, Bhatti AA (2017). E-waste flows, resource recovery and improvement of legal framework in Pakistan. Resour Conserv Recycl.

[CR30] Iqbal M, Breivik K, Syed JH, Malik RN, Li J, Zhang G, Jones KC (2015). Emerging issue of e-waste in Pakistan: a review of status, research needs and data gaps. Environ Pollut.

[CR31] Işıldar A, van de Vossenberg J, Rene ER, van Hullebusch ED, Lens PNL (2016). Two-step bioleaching of copper and gold from discarded printed circuit boards (PCB). Waste Manag.

[CR32] ISS (2015) Banca dati ISS - INAIL per Analisi di Rischio Sanitario – Ambientale (Marzo 2015)

[CR33] Itai T, Otsuka M, Asante KA, Muto M, Opoku-Ankomah Y, Ansa-Asare OD, Tanabe S (2014). Variation and distribution of metals and metalloids in soil/ash mixtures from Agbogbloshie e-waste recycling site in Accra, Ghana. Sci Total Environ.

[CR34] Jena PR, De S, Basu JK (2003). A generalized shrinking core model applied to batch adsorption. Chem Eng J.

[CR35] Jing-ying L, Xiu-li X, Wen-quan L (2012). Thiourea leaching gold and silver from the printed circuit boards of waste mobile phones. Waste Manag.

[CR36] Kasper AC, Berselli GB, Freitas BD, Tenorio JA, Bernardes AM, Veit HM (2011). Printed wiring boards for mobile phones: characterization and recycling of copper. Waste Manag.

[CR37] Kaya M (2016). Recovery of metals and nonmetals from electronic waste by physical and chemical recycling processes. Waste Manag.

[CR38] Kim E, Kim M, Lee J, Pandey BD (2011). Selective recovery of gold from waste mobile phone PCBs by hydrometallurgical process. J Hazard Mater.

[CR39] Kumar A, Holuszko ME, Janke T (2018). Characterization of the non-metal fraction of the processed waste printed circuit boards. Waste Manag.

[CR40] Labunska I, Abdallah MA-E, Eulaers I, Covaci A, Tao F, Wang M, Santillo D, Johnston P, Harrad S (2015). Human dietary intake of organohalogen contaminants at e-waste recycling sites in Eastern China. Environ Int.

[CR41] Levenspiel O (1999). Chemical reaction engineering.

[CR42] Li H, Yu L, Sheng G, Fu J, Peng P' (2007). Severe PCDD/F and PBDD/F pollution in air around an electronic waste dismantling area in China. Environ Sci Technol.

[CR43] Lu Y, Xu Z (2016). Precious metals recovery from waste printed circuit boards: a review for current status and perspective. Resour Conserv Recycl.

[CR44] Lundgren K (2012) The global impact of e-waste: addressing the challenge. International Labour Office, Programme on Safety and Health at Work and the Environment (SafeWork), Sectoral Activities Department (SECTOR). Geneva, ILO, 2012

[CR45] Moltó J, Egea S, Conesa JA, Font R (2011). Thermal decomposition of electronic wastes: Mobile phone case and other parts. Waste Manag.

[CR46] Oguchi M, Murakami S, Sakanakura H, Kida A, Kameya T (2011). A preliminary categorization of end-of-life electrical and electronic equipment as secondary metal resources. Waste Manag.

[CR47] Ohajinwa CM, Van Bodegom PM, Vijver MG, Peijnenburg WJGM (2017). Health risks awareness of electronic waste workers in the informal sector in Nigeria. Int J Environ Res Public Health.

[CR48] Ortuño N, Moltó J, Egea S, Font R, Conesa JA (2013). Thermogravimetric study of the decomposition of printed circuit boards from mobile phones. J Anal Appl Pyrolysis.

[CR49] Perkins DN, Brune Drisse M-N, Nxele T, Sly PD (2014). E-waste: a global hazard. Ann Glob Health.

[CR50] Sadhukhan AK, Gupta P, Saha RK (2010). Modelling of combustion characteristics of high ash coal char particles at high pressure: shrinking reactive core model. Fuel.

[CR51] Sepúlveda A, Schluep M, Renaud FG, Streicher M, Kuehr R, Hagelüken C, Gerecke AC (2010). A review of the environmental fate and effects of hazardous substances released from electrical and electronic equipments during recycling: examples from China and India. Environ Impact Assess Rev.

[CR52] Shibamoto T, Yasuhara A, Katami T (2007). Dioxin formation from waste incineration. Rev Environ Contam Toxicol.

[CR53] Song Q, Li J (2014). A systematic review of the human body burden of e-waste exposure in China. Environ Int.

[CR54] Steiner S (2004) Risk assessment of e-waste burning in Dehli, India. Swiss Federal Institute of Technology in Zurich (Switzerland). Thesis

[CR55] Stewart AG, Hursthouse AS (2018). Environment and human health: the challenge of uncertainty in risk assessment. Geosciences.

[CR56] Suresh SS, Mohanty S, Nayak SK (2017). Composition analysis and characterization of waste polyvinyl chloride (PVC) recovered from data cables. Waste Manag.

[CR57] Suzuki G, Someya M, Matsukami H, Tue NM, Uchida N, Tuyen LH, Viet PH, Takahashi S, Tanabe S, Brouwer A, Takigami H (2016). Comprehensive evaluation of dioxins and dioxin-like compounds in surface soils and river sediments from e-waste-processing sites in a village in northern Vietnam: heading towards the environmentally sound management of e-waste. Emerg Contam.

[CR58] Tansel B (2017). From electronic consumer products to e-wastes: global outlook, waste quantities, recycling challenges. Environ Int.

[CR59] Tsydenova O, Bengtsson M (2011). Chemical hazards associated with treatment of waste electrical and electronic equipment. Waste Manag.

[CR60] Tue NM, Goto A, Takahashi S, Itai T, Asante KA, Kunisue T, Tanabe S (2016). Release of chlorinated, brominated and mixed halogenated dioxin-related compounds to soils from open burning of e-waste in Agbogbloshie (Accra, Ghana). J Hazard Mater.

[CR61] US-EPA - Region 9 (2015). Environmental Protection Agency, Toxicity and chemical/physical properties for Regional Screening level (RSL) of Chemical Contaminants at Superfund Sites. http://www.epa.gov/region9/superfund/prg/

[CR62] US-EPA (2017) Regional screening levels-generic Tables (November 2017). https://www.epa.gov/risk/regional-screening-levels-rsls-generic-tables-november-2017. Accessed Nov 2017

[CR63] Veit HM, Juchneski NC de F, Scherer J (2014). Use of gravity separation in metals concentration from printed circuit board scraps. Rem Rev Esc Minas.

[CR64] Wang R, Xu Z (2014). Recycling of non-metallic fractions from waste electrical and electronic equipment (WEEE): a review. Waste Manag.

[CR65] Wang G, Wen Z, Lou G, Dou R, Li X, Liu X, Su F (2016). Mathematical modeling and combustion characteristic evaluation of a flue gas recirculation iron ore sintering process. Int J Heat Mass Transf.

[CR66] Williams PT (2010). Valorization of printed circuit boards from waste electrical and electronic equipment by pyrolysis. Waste Biomass Valoris.

[CR67] Wittsiepe J, Fobil JN, Till H, Burchard GD, Wilhelm M, Feldt T (2015). Levels of polychlorinated dibenzo-p-dioxins, dibenzofurans (PCDD/Fs) and biphenyls (PCBs) in blood of informal e-waste recycling workers from Agbogbloshie, Ghana, and controls. Environ Int.

[CR68] Wong MH, Wu SC, Deng WJ, Yu XZ, Luo Q, Leung AOW, Wong CSC, Luksemburg WJ, Wong AS (2007). Export of toxic chemicals—a review of the case of uncontrolled electronic-waste recycling. Environ Pollut.

[CR69] Yamane LH, de Moraes VT, Espinosa DCR, Tenório JAS (2011). Recycling of WEEE: characterization of spent printed circuit boards from mobile phones and computers. Waste Manag.

[CR70] Yazici EY, Deveci H (2013). Extraction of metals from waste printed circuit boards (WPCBs) in H_2_SO_4_-CuSO_4_-NaCl solutions. Hydrometallurgy.

[CR71] Yu J, Sun L, Wang B, Qiao Y, Xiang J, Hu S, Yao H (2016). Study on the behavior of heavy metals during thermal treatment of municipal solid waste (MSW) components. Environ Sci Pollut Res.

[CR72] Zeng X, Li J, Stevels ALN, Liu L (2013). Perspective of electronic waste management in China based on a legislation comparison between China and the EU. J Clean Prod.

[CR73] Zhang S, Forssberg E (1997). Mechanical separation-oriented characterization of electronic scrap. Resour Conserv Recycl.

[CR74] Zhang K, Pei Y, Lin C (2010). An investigation of correlations between different environmental assessments and risk assessment. Procedia Environ Sci.

[CR75] Zhang M, Buekens A, Jiang X, Li X (2015). Dioxins and polyvinylchloride in combustion and fires. Waste Manag Res.

[CR76] Zhao JP, Loo CE, Dukino RD (2015). Modelling fuel combustion in iron ore sintering. Combust Flame.

[CR77] Zhao J, Loo CE, Zhou H, Yuan J, Li X, Zhu Y, Yang G (2018). Modelling and analysis of the combustion behaviour of granulated fuel particles in iron ore sintering. Combust Flame.

[CR78] Zheng Y, Shen Z, Ma S, Cai C, Zhao X, Xing Y (2009). A novel approach to recycling of glass fibers from nonmetal materials of waste printed circuit boards. J Hazard Mater.

